# Protein Acetylation/Deacetylation: A Potential Strategy for Fungal Infection Control

**DOI:** 10.3389/fmicb.2020.574736

**Published:** 2020-10-07

**Authors:** Junzhu Chen, Qiong Liu, Lingbing Zeng, Xiaotian Huang

**Affiliations:** ^1^Department of Medical Microbiology, School of Medicine, Nanchang University, Nanchang, China; ^2^The First Affiliated Hospital of Nanchang University, Nanchang, China

**Keywords:** protein acetylation, protein deacetylation, fungal infection, virulence, KDAC inhibitors

## Abstract

Protein acetylation is a universal post-translational modification that fine-tunes the major cellular processes of many life forms. Although the mechanisms regulating protein acetylation have not been fully elucidated, this modification is finely tuned by both enzymatic and non-enzymatic mechanisms. Protein deacetylation is the reverse process of acetylation and is mediated by deacetylases. Together, protein acetylation and deacetylation constitute a reversible regulatory protein acetylation network. The recent application of mass spectrometry-based proteomics has led to accumulating evidence indicating that reversible protein acetylation may be related to fungal virulence because a substantial amount of virulence factors are acetylated. Additionally, the relationship between protein acetylation/deacetylation and fungal drug resistance has also been proven and the potential of deacetylase inhibitors as an anti-infective treatment has attracted attention. This review aimed to summarize the research progress in understanding fungal protein acetylation/deacetylation and discuss the mechanism of its mediation in fungal virulence, providing novel targets for the treatment of fungal infection.

## Introduction

The post-translational modification (PTM) of proteins is a major regulatory mechanism in all life forms. PTM refers to the chemical modification of amino acid residues in proteins by the addition of different chemical groups, which confer new properties on modified proteins, including changes in enzyme activity, subcellular localization, interaction partners, protein stability, and DNA binding (Mann and Jensen, 2003; Verdin and Ott, 2015). Currently, nearly 200 different types of PTMs have been identified, including acetylation, phosphorylation, alkylation, methylation, ubiquitination, and glycosylation (Garavelli, 2004). Protein acetylation, which refers to the covalent binding of an acetyl group to an amino acid residue of a protein, is the most well-known PTM besides phosphorylation (Ali et al., 2018). The most widely studied protein acetylation is that of lysine residues, although acetylation of serine and threonine side chains has also been reported (Tang and Yu, 2019). Thus, unless otherwise specified, in this review, acetylation refers only to that of lysine residues.

The acetyl group can be attached to the α-amino group on the N-terminal end of the protein or the ε-amino group on the side chain of lysine residues; therefore, acetylation can be classified as N^α^ -acetylation or N^ε^ -acetylation (Hentchel and Escalante-Semerena, 2015). At present, two mechanisms that can regulate acetylation have been identified: one mechanism is mainly regulated by lysine acetyltransferases (KATs), while the other mechanism is non-enzymatic, which can directly introduce lysine residues through the non-enzymatic reaction of acetyl phosphate or acetyl-CoA (Wagner and Hirschey, 2014; Lee et al., 2018). In prokaryotes, acetyl phosphate can modulate bacterial virulence through non-enzymatic acetylation (Ren et al., 2019). The main contributor of non-enzymatic acetylation in eukaryotes may be acetyl-CoA in the mitochondria; however, its role still needs to be studied further and is not the focus of this review (Weinert et al., 2014).

Protein deacetylation is the reverse reaction of acetylation that is catalyzed by lysine deacetylases (KDACs), which consist of two protein families, namely, classical Zn^2+^-dependent histone deacetylases (HDACs) and NAD^+^-dependent sirtuins (Table 1; Narita et al., 2019). Although acetylation occurs in an enzymatic or non-enzymatic manner, removal of the acetyl group requires KDACs. KDACs play a vital role in numerous biological processes by allowing chromatin condensation, thereby inhibiting transcription (Rupert et al., 2016). Many eukaryotic KATs and KDACs were initially identified as histone-specific enzymes and were historically named histone acetyltransferases and HDACs. In this review, we uniformly used the more specific terms KATs and KDACs.

**TABLE 1:** Some lysine acetyltransferases (KATs) and lysine deacetylases (KDACs) in fungi.

	KATs			KDACs			References
		
Classes	Gcn5 family	MYST family	Others	I	II	III	
*Candida albicans*	Gcn5	Esa1,Sas2, Sas3	Rtt109, Hat1, Elp3, Hpa2	Rpd31,Rpd32, Hos1, Hos2	Hda1,Hos3	Sir2, Hst1, Hst2, Hst3	Garnaud et al., 2016; Kim et al., 2018
*Saccharomyces cerevisiae*	Gcn5	Esa1, Sas2, Sas3	Rtt109, Hat1, Elp3, Hpa2, Hpa3	Rpd3, Hos1, Hos2	Hda1, Hos3	Sir2, Hst1, Hst2, Hst3, Hst4	Garnaud et al., 2016; Kim et al., 2018
*Cryptococcus neoformans*	Gcn5			Rpd3, Hos1, Hos2, Cir61, Cir62	Hda1, Hos3	Sir2, Hst1, Hst2, Hst3, Hst4, Hst5	O’Meara et al., 2010; Wassano et al., 2020
*Fusarium graminearum*	Gcn5	Sas2, Sas3	Rtt109, Elp3	Rpd3, Hos2	Hda1, Hos3		Li et al., 2011; Kong et al., 2018
*Aspergillus nidulans*	GcnE	EsaA		HosA	HdaA	SirA	Tribus et al., 2005; Reyes-Dominguez et al., 2008; Soukup et al., 2012; Itoh et al., 2017; Pidroni et al., 2018
*Magnaporthe oryzae*	Gcn5	Sas3	Rtt109, Hat1	Hos2	Hda1		Maeda et al., 2017; Kwon et al., 2018; Dubey et al., 2019; Lee et al., 2019; Yin et al., 2019
*Aspergillus fumigatus*	GcnE		Rtt109	RpdA, HosA	HdaA, HosB	SirA, SirB, SirE, SirC, SirD, HstA	Graessle et al., 2000; Lee et al., 2009; Bauer et al., 2019; Lin et al., 2020; Wassano et al., 2020

*The KATs and KDACs listed in table do not represent all the KATs and KDACs of the microbe. KAT, lysine acetyltransferase; KDAC, lysine deacetylase; MYST family, Moz, Ybf2/Sas3, Sas2, and Tip family.*

A substantial amount of evidence showed that acetylation and deacetylation play essential roles in modifying the chromosome structure and regulating gene expression (Nicolas et al., 2018). Acetylation and deacetylation can also modify many key cellular processes relevant to physiology and disease, such as enzymatic activity, signal transduction, DNA damage repair, cell division, metabolism, autophagy, protein stability, and protein localization and interactions (Eckschlager et al., 2017; Narita et al., 2019). Hence, protein acetylation and deacetylation can interfere with every step in a regulatory process, thereby altering cell fate and function.

Research on acetylation/deacetylation is currently focused on metabolism, tumor treatment, and other aspects, while there is less research on microbial acetylation, especially regarding microbial virulence. The development of proteomics has resulted in accumulating evidence that protein acetylation/deacetylation is related to microbial virulence and drug resistance (Hnisz et al., 2010; Li et al., 2017; Brandão et al., 2018; Ren et al., 2019). The role of acetylation in regulating bacterial virulence was summarized in a review conducted by Ren et al. (2017). However, the relationship between the specific mechanism of acetylation and fungal pathogenicity remains unclear.

Fungal pathogens have a negative impact on the global economy, food security, and human and animal welfare, not only because they have caused pestilence and famine but also because of the difficulty in treating fungal infectious diseases as well as increased resistance to antifungal drugs (Fisher et al., 2016; Motaung et al., 2017). In animals and plants, an unprecedented number of fungal and fungal-like diseases have led to some of the most serious deaths and extinctions in wild species (Fisher et al., 2016). Therefore, the virulence of pathogenic fungi must be explored. This review discusses how acetylation/deacetylation regulates fungal virulence. First, we discussed the widespread distribution of this modification in the fungal community and listed some virulence-related acetylated proteins present in fungi. Then, we highlighted recent examples to illustrate the unexpected role of acetylation/deacetylation in fungal virulence to suggest novel targets for the development of anti-infective drugs and the treatment of infectious diseases.

### Acetylation/Deacetylation is Widespread in Fungi

Protein acetylation is a conserved evolutionary modification that occurs in eukaryotic and prokaryotic proteins and was first discovered in histones (Phillips, 1963; Allfrey et al., 1964). Reversible protein acetylation was studied in the context of the histones until the late 1990s. Recent advancements in high-resolution mass spectrometry and high-affinity purification technology for acetylated lysine peptides revealed that protein acetylation/deacetylation is not restricted to histones, which resulted in detailed studies of the acetylated proteome and its function (Schilling et al., 2019).

Previous studies and database searches revealed that protein acetylation is widespread in fungi. Zhou et al. detected 477 acetylated proteins (5.28%) among all 9,038 proteins of *Candida albicans*, which was the first study on acetylome in human pathogenic fungi, providing an important initiating point for further study of the functional analysis of acetylated proteins in such fungal pathogens (Zhou X. et al., 2016). The comparative analysis of fungal acetylomes plays an important role in determining the essential role of acetylation in the virulence of human fungal pathogens (Li et al., 2019). Significant differences in the number and sites of acetylated proteins were found according to the stage of human fungal pathogen growth. For example, 2,335 proteins in the mycelium growing stage were identified to be acetylated in *Trichophyton rubrum*, which was >10 times higher than that in the conidia stage, and may be explained by conidia being in a quiescent state with low metabolic activity (Xu et al., 2018). Further evidence of protein acetylation in human fungal pathogens was observed in *Histoplasma capsulatum*, *Cryptococcus neoformans*, and *Aspergillus fumigatus* (Xie et al., 2016; Brandão et al., 2018; Lin et al., 2020).

In plant pathogenic fungi, Yang et al. identified 1,313 high-confidence acetylation sites in 727 acetylated proteins in *Aspergillus flavus*, while 577 acetylated sites were reported in 364 different proteins in *Fusarium graminearum* (Zhou S. et al., 2016; Yang et al., 2019). Several published studies have described the acetylome of different fungal species, including plant pathogenic fungi *Phytophthora sojae*, *Botrytis cinerea*, and *Magnaporthe oryzae*; fungal insect pathogens, such as *Beauveria bassiana* and *Metarhizium anisopliae*; and nonpathogenic fungi species, such as *Saccharomyces cerevisiae* and *Yarrowia lipolytica*; which are considered as important resources to explore the physiological role of this modification in eukaryotes (Henriksen et al., 2012; Mukherjee et al., 2012; Li et al., 2016; Lv et al., 2016; Wang et al., 2017; Cai et al., 2018c; Liang et al., 2018).

Interestingly, most of the identified acetylated proteins were involved in the regulation of glucose, lipid, and amino acid metabolism (Wang et al., 2017). Important findings regarding the control of metabolism via protein acetylation were reported in prokaryotes (Wang et al., 2010). A large number of metabolic enzymes are also acetylated in *S. cerevisiae*, which is consistent with the enzymes that regulate central metabolism through reversible acetylation, ensuring that cells respond to environmental changes by rapidly sensing the cellular energy state and flexibly changing rates or direction (Wang et al., 2010; Henriksen et al., 2012). This result can be explained by the central role of acetyl-CoA in intermediary metabolism because acetyl-CoA acts as the acetyl-donor for both enzymatic and non-enzymatic acetylation (Trefely et al., 2019). In other words, the dynamic interplay between cellular metabolism and acetylation plays a key role in epigenetics; however, this is not the focus of this review.

In summary, protein acetylation is widely distributed in fungi. Aside from modifying many key cellular processes, such as enzymatic activity, signal transduction, cell division, and metabolism, it also controls morphological transformation, biofilm formation, acetic acid stress tolerance, and other processes, thereby affecting the entire fungal life cycle (Kim et al., 2015; Cheng et al., 2016; Narita et al., 2019; Lin et al., 2020).

## Role of Acetylation/Deacetylation in Fungal Virulence

All known bacterial KATs that have been discovered to date belong to the Gcn5-related N-acetyltransferase family; fewer deacetylases are encoded by prokaryotes, which means that acetylation and deacetylation processes in fungi are more complex with a higher proportion of acetylated proteins in eukaryotes (Tables 1, 2; Hentchel and Escalante-Semerena, 2015). KATs play a vital role in the morphogenetic hyphae growth, biofilm formation, drug resistance, and virulence (Kong et al., 2018; Lin et al., 2020). In *B. bassiana*, deletion of g*cn5* led to severe defects in colony growth and loss of cuticle infection (Cai et al., 2018b). In *P. sojae*, although Δ*gcn5* mutants had a normal development, their virulence in soybean was significantly reduced (Zhao et al., 2015). KDACs are also necessary for fungal pathogenesis, which were found to be decisive regulators of genes involved in pathogenicity and fungal toxin production, regulating a number of physiological processes, including thermotolerance, capsule formation, melanin synthesis, protease activity, and cell wall integrity (Bauer et al., 2016, 2019; Brandão et al., 2018).

**TABLE 2:** Some representative acetylated proteins in fungi.

Species	Acetylated protein	Function	References
*Candida albicans*	Hsp90	Regulates stress responses and cellular signaling; mediates azole resistance.	Li et al., 2017
*Saccharomyces cerevisiae*	Pck1p	Controls prompt adaptation of a metabolic flux to energy status.	Lin et al., 2009
	Smc3p	Affects cohesion establishment.	Heidinger-Pauli et al., 2010
	Hsp90	Regulates stress responses and cellular signaling; mediates azole resistance.	Robbins et al., 2012
*Aspergillus flavus*	AflO	Affects aflatoxin production and pathogenicity.	Yang et al., 2019
*Aspergillus fumigatus*	Hsp90	Regulates drug resistance.	Lamoth et al., 2014
	CBP	Involved in intracellular Ca^2+^ signaling.	Xie et al., 2016
*Histoplasma capsulatum*	Hsp60	Interacts with CR3 molecules on host phagocytes; involved in *Histoplasma* attachment to host macrophages.	
	Hsp70	Implicated in microbial virulence.	
*Magnaporthe oryzae*	Atg3	Involved in autophagy during both appressorium development and nutrient starvation.	Yin et al., 2019
	Atg9	Affects development and pathogenicity of *M. oryzae.*	
*Fusarium graminearum*	FgFkbp12	Rapamycin toxicity.	Zhou and Wu, 2019
	FaTUA1	Virulence, hyphae growth.	
	GzOB031	Virulence.	
	GzBrom002	DON, virulence, sexual and asexual.	
	FCA6	Peroxidase activities.	
	PKR	DON, virulence, sexual and asexual.	
*Trichophyton rubrum*	Hsp90	Regulates drug resistance and growth in human nails *in vitro*.	Jacob et al., 2015

Many fungal phenotypes have shown a specific correlation with virulence, such as biofilm formation, capsule production, melanin formation, and the secretion of various proteins (Staniszewska et al., 2012; Alspaugh, 2015). Additionally, cellular features, such as the cell wall, hyphae formation, stress response and morphological transition, allow the rapid and effective adaptation of fungal pathogens to varying conditions, which is conducive to their survival in the environment and in infected hosts (Wang et al., 2013; Alspaugh, 2015; Kim et al., 2015). Here, we primarily focused on the KATs and KDACs to discuss the role of acetylation and deacetylation in fungal virulence (Table 1), particularly in *A. fumigatus*, *C. neoformans*, and *C. albicans*, which are important clinical and useful research models for studying fatal infectious fungal pathogens in humans.

### Acetylation/Deacetylation Regulates Fungal Stress Response

Generally, pathogens are subjected to various environmental challenges, such as temperature variations, an acidic pH, and oxidative stress. Reversible acetylation has emerged as one of the processes critical to maintaining cellular homeostasis and shaping responses to environmental stimuli (O’Meara et al., 2010; Chang et al., 2015; Wang J.-J. et al., 2018). In *C. neoformans*, the loss of acetylation gene *gcn5* caused a reduction in toxicity in a murine intranasal infection model and growth defects at high temperatures (O’Meara et al., 2010). In *B. bassiana*, the deletion of *gcn5* led to a 97% reduction in the conidiation capacity as well as severe defects in the growth of fungal colonies and conidial thermotolerance (Cai et al., 2018b). Moreover, Mst2, which can specifically acetylate histone H3K14 through cooperation with Gcn5 to regulate global acetylation events in *B. bassiana*, was found to play an important role in sustaining multiple stress tolerances such as osmotic and oxidative stress tolerance, cell wall perturbing stress tolerance, thermotolerance, and UV-B resistance (Wang J.-J. et al., 2018). Furthermore, the Δ*gcn5* mutants of *S. cerevisiae* and *Schizosaccharomyces pombe* showed defects in the cellular response to many stressors, including elevated temperatures, high salt concentrations, and nutrient deprivation (Chang et al., 2015).

The absence of acetyltransferase Rtt109 in *S. cerevisiae* not only activated the transcription of stress-responsive genes but also improved the resistance to oxidative stress, which ultimately contributed to the improvement in acetic acid tolerance (Cheng et al., 2016). The KDAC sirtuin 2, played a role in starvation stress resistance in yeasts; the deacetylase gene *rpd3* was also considered essential for starvation stress resistance (Fulco et al., 2003; Nakajima et al., 2016). A previous study on *B. bassiana* suggested that Δ*rpd3* significantly reduced the conidial tolerance to wet-heat stress at 45°C but increased the conidial resistance to UV-B irradiation, and the fungal virulence was greatly attenuated in the absence of *rpd3* (Cai et al., 2018c). Studies have found that the downregulation of RPD3-type deacetylase RpdA leads to avirulence of *A. fumigatus* in a murine model for pulmonary aspergillosis (Bauer et al., 2019). In addition, KDACs also play a decisive role as virulence factors in the pathogenic fungus *Cochliobolus carbonum* and *B. bassiana* (Baidyaroy et al., 2001; Zhang et al., 2020). Although the mechanism of virulence attenuation and adverse environmental tolerance remains unclear, previous evidence suggests that acetylation/deacetylation can control the virulence level of pathogens by regulating their stress response.

### Acetylation/Deacetylation Rregulates Hyphal Growth

Hyphae have a strong ability to adhere and invade the host, making it easy to maintain their colonization and escape attacks from the host immune system, probably through the release of cell type-specific virulence factors, such as adhesins (e.g., Hwp1, Als3, Als10, Fav2, and Pga55), tissue-degrading enzymes (e.g., Sap4, Sap5, and Sap6), and antioxidant defense proteins (e.g., Sod5) (Sudbery, 2011; Noble et al., 2017). Acetylation and deacetylation play critical regulatory roles in regulating the initiation and maintenance of hyphal development (Garnaud et al., 2016; Kong et al., 2018; Lee et al., 2019). Tribus et al. (2010) found that the repression of the promoter of *rpdA* knockdown strains resulted in distorted and hyperbranched hyphae and a tremendous loss of radial growth of fungal colonies. MoHOS2-mediated histone deacetylation is important for the development of *M. oryzae*. In the absence of this mechanism, *M. oryzae* exhibits defects in hyphae formation, thereby impairing its growth ability inside the host plant (Lee et al., 2019). In *B. bassiana*, the hyphal cells of Δ*hos2* mutants are significantly longer than those of the wild type strains, which was concurrent with its inability to develop intact nuclei in hyphal cells (Cai et al., 2018a). The hyphal growth defects of four acetyltransferase mutants of *F. graminearum*, namely, Δ*FgGCN5*, Δ*FgRTT109*, Δ*FgSAS2*, and Δ*FgSAS3* mutants in solid medium, have also been reported (Kong et al., 2018).

In *C. albicans*, deacetylase Rpd31 and Set3C (Set3/Hos2 HDAC complex) are crucial repressors of the yeast-to-hyphae transition in *C. albicans* (Hnisz et al., 2010; Garnaud et al., 2016). The acetyltransferase activity of nucleosome acetyltransferase of H4 (NuA4) and the deacetylase activity of Hda1 have also been reported as essential for hyphal initiation and maintenance (Wang X. et al., 2018). Furthermore, NuA4 dynamically regulates hyphal growth by merging and separating with the SWR1 complex, which was mediated by the acetylation of Eaf1 at K173 (lysine residue 173) (Wang X. et al., 2018). Gcn5 was also required for the invasive and filamentous growth of *C. albicans*, while *gcn5* mutant impaired the hyphal elongation in sensing serum and attenuated the *C. albicans* virulence in a mouse systemic infection model (Chang et al., 2015). Further evidence of the association between acetylation and hyphal growth was found in the acetyltransferase Esa1, which belongs to the MYST (Moz, YBF2, Sas2p, and Tip) family. Wang et al. found that *Esa1* was not important for the general growth of *C. albicans* but was important for its filamentous growth and that *Esa1* deletion could prevent filament formation under all hyphal induction conditions (Wang et al., 2013). Overall, hyphal initiation, development, and maintenance are complex processes regulated by acetylation and deacetylation in both filamentous fungi and budding yeasts.

### Acetylation/Deacetylation Regulates Morphological Transition

**FIGURE 1:**
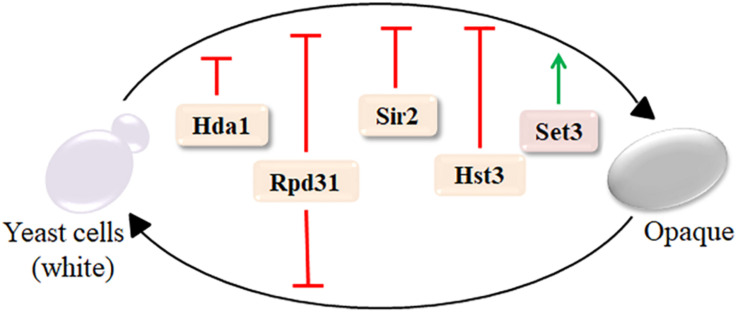
Lysine deacetylases (KDACs) regulate the white-to-opaque switch in *C. albicans*. Set3 only activates the white-to-opaque switch; Hda1, Sir2, and Hst3 inhibit the progress; and Rpd31 suppresses transitions in both directions. Green arrows indicate activation, while red lines indicate inhibitory functions. Reproduced with the kind permission from Frontiers in Microbiology (Garnaud et al., 2016) with adjusted color scheme.

One of the key virulence traits of fungi is morphological plasticity (Li et al., 2017). Although some human fungal pathogens mainly exist in the form of budding yeast cells (such as *C. neoformans*) or filamentous hyphal structures (such as *Aspergillus*), *C. albicans* alternates between these and other forms, usually in response to specific environmental cues (Takagi et al., 2019). In addition to yeast-to-hyphae transition, *C. albicans* can undergo a reversible switch between two morphologies, known as the white and opaque phases. Although the white and opaque cell types share the same genome, white cells caused more severe virulent in toxicity in mouse models (Kvaal et al., 1997). The class II deacetylase Hda1 selectively inhibits white-to-opaque switches, while the class I deacetylase Rpd31 suppresses transitions in both directions (Srikantha et al., 2001). Moreover, the sirtuins Hst3 and Sir2 were repressors of the white-to-opaque switch, whereas Set3C was the key activator (Figure 1; Pérez-Martín et al., 1999; Hnisz et al., 2009; Stevenson and Liu, 2011). Thus, *C. albicans* requires interaction with KDACs function for its morphological plasticity, which is central to its pathogenesis.

### Acetylation/Deacetylation Regulates Biofilm Formation

Biofilm formation on host tissues and indwelling medical devices is highly associated with fungal pathogenicity and drug resistance because the extracellular matrix hinders drug diffusion (Nobile et al., 2014). Fungal adhesion on both biotic and abiotic surfaces is the first phase of biofilm formation, which is closely related to the fungal cell wall and is critical to all later stages of biofilm development (Nett et al., 2011; Lohse et al., 2018). The relationship between biofilm resistance and the cell wall integrity pathway has been confirmed (Nett et al., 2011). In *C. albicans*, Nobile et al. (2014) found that the deletion of *set3* and *hos2* in *C. albicans* reduced biofilm formation and biomass, and these mutants appeared more resistant to yeast dispersion *in vivo*. Heat shock protein 90 (Hsp90) was a key regulator of biofilm dispersion and drug resistance and could be acetylated on lysine 27 and 270 (Robbins et al., 2012). Compromised Hsp90 function reduced the biofilm formation of *C. albicans in vitro* and impaired the dispersal of biofilm cells, blocking their capacity to serve as reservoirs of infection (Robbins et al., 2011). Moreover, Hsp90 was involved in the resistance of *A. fumigatus* biofilms to drugs (Robbins et al., 2011). Another study on *A. fumigatus* found that acetyltransferase GcnE was also required for biofilm formation (Lin et al., 2020). The list of device-associated infections caused by biofilms is expanding daily. Thus, the urgent determination of the mechanisms whereby acetylation/deacetylation participates in regulating biofilm formation is crucial.

### Acetylation/Deacetylation Regulates Secondary Metabolite Production

A distinguishing feature of fungi is their ability to produce a variety of small molecules that contribute to their survival and pathogenicity. These substances include compounds such as pigments, which play a role in virulence and protect fungi from environmental damage, and toxins that kill host tissues or hinder competition from other organisms. The absence of deacetylase HdaA in *Aspergillus nidulans* caused the upregulation of carcinogenic sterigmatocystin (Shwab et al., 2007). In *A. fumigatus*, Δ*hdaA* knockout strains had a decreased production of the virulence factor gliotoxin (Lee et al., 2009). In the plant pathogenic fungus *Fusarium fujikuroi*, the deletion of *hda1* or *hda2* inhibited the production of red polyketide pigment bikaverin, plant hormone gibberellin, and mycotoxin fumaric acid; however, the deletion of *hda1* did not affect the production of mycotoxin fusarins, and the deletion of *hda2* did not affect the production of pigment fusarubin. This finding indicated that the impact of acetylation on transcriptional regulation is usually more complex because of the functional complementarity of different KDAC genes (Studt et al., 2013).

AflO, a key enzyme in aflatoxin biosynthesis, was acetylated at lysine 241 and 384 and played a vital role in the pathogenicity of *A. flavus* (Yang et al., 2019). Six proteins involved in the virulence of *B. cinerea* were found to be acetylated (BcSak1, Hpt1, Bcchs2, CHSV, PKS, and BOS1) (Lv et al., 2016). In *F. graminearum*, 10 virulence-related proteins were also acetylated, including Kin4, Sty1, and Gpmk1 (Zhou X. et al., 2016). Deoxynivalenol (DON), a mycotoxin produced by *F. graminearum*, is a virulence factor that helps fungi colonize and spread within spikes (Kong et al., 2018). The DON production levels of Δ*FgSAS3* and Δ*FgGCN5* mutants were almost zero compared with that of wild type strain (Kong et al., 2018). Although DON is not a protein, acetylation plays an important role in its metabolism.

Melanin is a pigmented polymer that protects fungal cells against oxidative stress, phagocytosis, and antifungal drugs. It also modifies the host immune responses by reducing the susceptibility of melanized microbes to the host defense mechanisms (Brandão et al., 2015, 2018). Brandão et al. (2018) found that the change in virulence of Δ*hda1* mutants of *C. neoformans* might be due to its markedly reduced formation of capsule, melanin, and extracellular proteases, all of which are specifically required for the survival of microbes in the host. Maeda et al. (2017) found that deletion of the *HdaA* homolog in *Magnaporthe orzyae* increased the expression of melanin biosynthesis genes. Although the effect of KDAC inhibitors (KDACis) on melanin synthesis has been confirmed, the specific mechanism of acetylation in melanin formation remains unclear (Brandão et al., 2015). The abovementioned studies suggest that protein acetylation/deacetylation can affect the virulence of fungi by participating in the regulation of secondary metabolite biosynthesis.

## Application of Acetylation/Deacetylation in Antifungal Therapy

**FIGURE 2:**
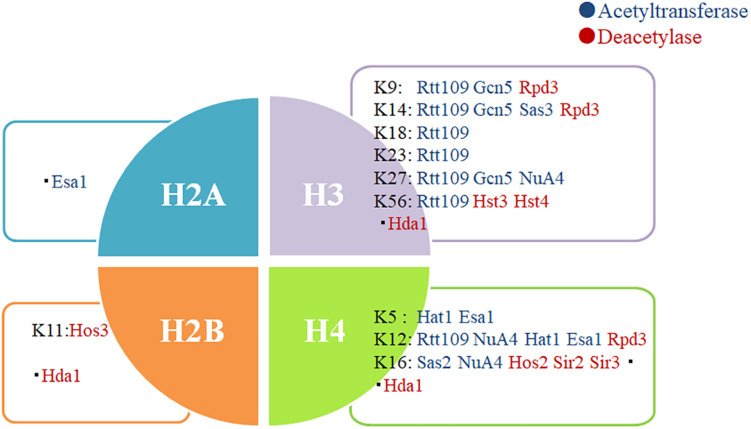
Some important acetylation sites of histones. Protein acetylation on the nucleosomal histones is essential in the regulation of chromatin structure and expression of genes. Some histone acetylation sites that play an important role in fungi have been summarized. A few studies have reported the action sites of Esa1 and Hda1 on histones.

Invasive infections caused by fungal pathogens are a major public health issue. More than 1.6 million people worldwide develop serious fungal diseases that have a major or fatal impact on their lives (Bongomin et al., 2017). Although the development of new antifungal drugs is an important strategy for the treatment of fungal infections, the urgent development of new infection treatment strategies in view of the uncontrolled increase in the incidence of drug-resistant fungal infections worldwide is crucial (Perlin et al., 2017). Enzymes that control chromatin modification could form a new group of antimicrobial target genes because they are involved in many pathophysiological processes that regulate virulence (Tscherner et al., 2015; Bauer et al., 2016). These results may shed new light on KATs/KDACs as a potential therapeutic target for developing an anti-infection drug (Figure 2).

Because KDACis can regulate reversible protein acetylation by inhibiting KDAC activity, altering gene expression; inducing cell cycle arrest, cell differentiation, and apoptosis; reducing angiogenesis; and modulating immune response; they became a new hotspot in the research of tumor-targeted therapy (Spange et al., 2009; Eckschlager et al., 2017). Currently, KDACis are broadly classified into five main groups: hydroxamates, cyclic peptides, benzamides, short-chain fatty acids, and sirtuin inhibitors (Table 3; von Knethen and Brüne, 2019). At present, KDACis are widely used in clinical practice; however their application value in the treatment of antifungal infections requires further investigation.

**TABLE 3:** Classification of lysine deacetylase inhibitors.

Class	Name	KDAC specificity	Clinical trial stage
Hydroxamates	Suberoylanilide hydroxamic acid (SAHA)	Pan-KDACi	Approved in 2006 for CTCL
	Belinostat (PXD101)	Pan-KDACi	Approved in 2014 for PTCL
	Panobinostat (LBH-589)	Pan-KDACi	Approved in 2015 for MM
	Trichostatin A (TSA)	Pan-KDACi	Preclinical
	Givinostat (ITF2357)	Pan-KDACi	Phase II
	Resminostat (4SC201)	Pan-KDACi	Phase II
	Abexinostat (PCI24781	Pan-KDACi	Phase II
	Practinostat (SB939)	Class I, II, IV KDACi	Phase II
	Rocilinostat (ACY1215)	Class II KDACi	Phase I
	Pyroxamide (NSC696085)	HDAC1 inhibitor	Phase I
	CHR-3996	Class I KDACi	Phase I
	AR42	Pan-KDACi	Phase I
Cyclic peptides	Romidepsin (FK288)	Class I KDACi	Approved in 2009 for CTCL
Benzamides	Tacedinaline (CI994)	HDAC1-3 inhibitor	Phase III
	Entinostat (MS-275)	Class I KDACi	Phase II
	Mocetinostat (MGCD0103)	Class I, IV KDACi	Phase II
	4SC202	Class I KDACi	Phase I
Short chainfatty acids	Valproic acid	Class I, IIa KDACi	Phase III
	Phenylbutyric acid	Pan-KDACi	Phase II
	Butyric acid	Pan-KDAC inhibitor	Phase II
Sirtuin inhibitors	Cambinol	SIRT1,2 inhibitor	Preclinical
	Sirtinol	SIRT1,2 inhibitor	Preclinical
	EX-527	SIRT1,2 inhibitor	Phase I
	Nicotinamide (NAM)	Class III KDACi	Phase III

*KDAC, lysine deacetylase; KDACi, lysine deacetylase inhibitor; CTCL, cutaneous T-cell lymphoma; PTCL, peripheral T-cell lymphoma; MM, multiple myeloma.*

Targeting fungal deacetylases as a therapeutic strategy has a particular advantage. Inhibiting fungal KDACs may have beneficial and synergistic effects by reducing the virulence and growth of fungi, while also decreasing their tolerance and resistance to the existing antifungal drugs (Pfaller et al., 2015; Zhang and Xu, 2015). Previous studies found that KDACis could inhibit the production of toxic factors in *C. neoformans*; in *S. cerevisiae*, the deacetylase inhibitor trichostatin A (TSA) could eliminate its resistance to azole drugs by inhibiting the KDAC activity (Robbins et al., 2012; Brandão et al., 2015). Lys56 acetylation of histone H3 in *C. albicans* was an ideal target for antifungal therapy, and reduced levels of H3K56ac sensitized *C. albicans* to genotoxic and antifungal agents (Figure 2; Wurtele et al., 2010). In *A. fumigatus*, Hsp90 acetylation was also involved in the regulation of drug resistance (Lamoth et al., 2014). Deacetylases Hda1 and Rpd3 can regulate the function of Hsp90 to control fungal drug resistance; therefore, KDACis can block the emergence and maintenance of Hsp90-dependent azole resistance (Robbins et al., 2012). These studies demonstrate that KDACis hold great promise in the treatment of infections resistant to antifungal agents.

Little attention has been paid to acetylase inhibitors because KATs are rarely considered as drug targets. One of the reasons may be the promising application of KDACis in various diseases, and the other is that only a small number of acetylase inhibitors have been identified (Spange et al., 2009). Thus, we mainly discuss the application of KDACis in the treatment of fungal infections using several representative KDACis as examples.

### Pan-KDACis

TSA is the best-known broad-spectrum KDACi. It was first isolated from a culture medium of *Streptomyces platensis* and initially appeared as an antifungal drug to inhibit the growth of *Trichophyton* and *Aspergillus* (Tsuji et al., 1976). The inhibition of RpdA activity by TSA resulted in a significant delay in the growth and germination of fungal species, such as *A. fumigatus*, *A. nidulans, Aspergillus terreus, Penicillium chrysogenum*, and *Neurospora crassa* (Bauer et al., 2016). Hnisz et al. (2010) found that TSA was involved in triggering the yeast-to-hyphae conversion of *C. albicans* by inhibiting Set3C, which controls protein kinase A signaling through Efg1. TSA also increased the susceptibility of *Candida* sp. to azole antifungals by inhibiting the biosynthesis of ergosterol (Smith and Edlind, 2002). Lamoth et al. (2014) found that the combination of TSA and azole drugs in the treatment of *A. fumigatus* also showed a promising possibility. Considering instability and rapid metabolism of TSA, the development of highly selective inhibitors is very important for mitigating potential toxicities caused by high doses (Tan and Liu, 2015). Sodium butyrate, apicidin, and suberoylanilide hydroxamic acid are also effective broad-spectrum KDACis; however, their use in antifungal therapy requires further investigation.

### Selective KDACis

MGCD290, a fungal-specific Hos2 inhibitor in *Candida* sp., displayed moderate activity when used alone (Pfaller et al., 2009, 2015). However, the use of MGCD290 in combination with fluconazole, voriconazole, and posaconazole significantly increased the susceptibility of fungal species *in vitro* such as azole-resistant *Candida*, *Mucor*, and *Fusarium* sp. (Pfaller et al., 2009; Lamoth et al., 2015). When fluconazole, which had inactive activity against filamentous fungi, was used in combination with MGCD290, there was a distinctly favorable influence of the fluconazole MICs of *Aspergillus* strains, resulting in a conversion from resistance to susceptibility (Pfaller et al., 2009). Interestingly, *Hos2* was a homologous gene of *HosA* in *A. nidulans* (Pidroni et al., 2018). However, the deletion of *HosA* did not affect the efficacy of any antifungal drugs, which contradicted the specificity of MGCD290 (Pidroni et al., 2018). These contradictory results may be explained by the different biological functions of HosA-type proteins in different *Aspergillus* species, or more likely by the fact that MGCD290 does not specifically act on HosA-type enzymes in filamentous fungi (Pidroni et al., 2018). Therefore, although the specificity of MGCD290 is a debatable issue, MGCD290 has great application prospects in antifungal therapies.

### Sirtuin Inhibitors

Nicotinamide (NAM), a form of vitamin B3, is a typical non-competitive inhibitor of sirtuins (Orlandi et al., 2017). NAM possesses an antibacterial activity, inhibits cell proliferation and enhances the antiproliferative effect of cytostatic drugs (Xing et al., 2019). NAM’s potential to inhibit the growth of *Mycobacterium tuberculosis*, *Plasmodium falciparum*, and HIV has been demonstrated in clinical trials (Murray, 2003; Tcherniuk et al., 2017). Furthermore, NAM displayed broad-spectrum activity against multiple clinical isolates, including *C*. *parapsilosis*, *C*. *tropicalis*, *C*. *glabrata*, *C*. *krusei*, *C. neoformans*, and fluconazole-resistant *C. albicans* (Xing et al., 2019). NAM also reduced the kidney burden in a mouse model of disseminated candidiasis (Wurtele et al., 2010). Moreover, two different *Aspergillus* species, *A. fumigatus*, and *A. nidulans*, were very sensitive to NAM (Wurtele et al., 2010). NAM also reduced the activity of some enzymes produced by fungi, such as *C. albicans*, *T. rubrum*, and *Trichophyton mentagrophytes*, which supports the use of NAM as an antifungal drug (Ciebiada-Adamiec et al., 2010). In addition to NAM, sirtuin inhibitors include the specific SIRT1 and SIRT2 inhibitors sirtinol, cambinol and EX-527, but their value in the treatment of infection is unclear (Eckschlager et al., 2017).

## Outlook and Conclusion

As a common PTM, protein acetylation plays an essential role in metabolism, virulence, transcription, and translation, among other processes. Acetylation is primarily catalyzed by specific acetyltransferases but can also occur due to the non-enzymatic reactions of acetyl phosphates. At present, only a few studies have reported on the latter, and the relationship between the two different acetylation processes in the regulation of microbial virulence remains unclear. Furthermore, it is unknown whether other non-enzymatic/enzymatic acetylation mechanisms exist and how these (de)acetylation mechanisms cooperate.

Additionally, fungal virulence is a complex phenotype involving multiple factors, making it difficult to explain by analyzing a single type of PTM because there may be multiple PTMs on the same protein, and one protein usually has multiple acetylated lysine residues. How do multiple PTMs cooperate in response to different environmental changes? Besides acetylation, are there other types of acylation that affect the regulation of acetylation, such as malonylation, glutarylation, succinylation, methylation, propionylation, and butyrylation? Do these acetylation modifications have an effect on fungal virulence? These questions need to be addressed in future studies.

At present, research on acetylation and deacetylation mainly focuses on human metabolism, tumors, and other aspects. By contrast, studies on microbial acetylation/deacetylation are limited and mainly focus on a few microbial species, such as *Escherichia coli*, *Salmonella typhimurium*, *M. tuberculosis*, *S. cerevisiae*, and *C. albicans*; and studies on human fungal pathogens mainly focus on *C. albicans*, *C. neoformans*, and *A. fumigatus* (Brandão et al., 2018; Bauer et al., 2019; Lin et al., 2020). With the rapid development of protein detection technology, such as high-resolution mass spectrometry, and the broad application of the protein chip, developments in these fields will greatly enrich the investigations on the role of acetylation/deacetylation in regulating microbial physiological process, especially that of microbial pathogenesis and immunity. Therefore, further study of other pathogens is important to reveal the effect of protein acetylation/deacetylation on fungal toxicity and its potential mechanism and may provide some novel potential drug targets for drug development. Finally, in most cases, the effect of the regulation of protein acetylation in host cells by specific pathogens on the quality of immune responses to a broad range of pathogens has not been studied. Future investigations need to be rationally designed to analyze both the pathogen itself and the host’s immune status to avoid excessive damage to the host’s tissues.

## Author Contributions

All authors contributed to the critical analysis of the collected data and writing of the manuscript. All authors approved the final manuscript.

## Conflict of Interest

The authors declare that the research was conducted in the absence of any commercial or financial relationships that could be construed as a potential conflict of interest.
